# Adoption of positive health behaviour among primary care physicians: a cross-sectional pilot study from Lebanon

**DOI:** 10.3399/bjgpopen18X101590

**Published:** 2018-05-16

**Authors:** Rim Taleb, Issam Shaarani, Najla Lakkis, Rana El-Jarrah, Mona Osman

**Affiliations:** 1 Lecturer, Faculty of Medicine, Beirut Arab University, Beirut, Lebanon; 2 Senior Lecturer, Faculty of Medicine, Beirut Arab University, Beirut, Lebanon; 3 Clinical Associate, Department of Family Medicine, American University of Beirut Medical Center, Beirut, Lebanon; 4 Resident, Department of Family Medicine, American University of Beirut Medical Center, Beirut, Lebanon; 5 Instructor of Clinical Family Medicine, Department of Family Medicine, American University of Beirut Medical Center, Beirut, Lebanon

**Keywords:** Primary care physicians, health behaviour, preventive services, screening

## Background and method

In Lebanon, primary care physicians (PCPs), including family doctors and GPs, are the main healthcare providers at the primary care level. The difference between the two is that GPs practice medicine directly after medical school without additional specialisation, while family physicians complete a family medicine residency before starting practice. PCPs have an essential role in counselling and providing health education, which are integral components of comprehensive care.

Health behaviour includes practices, actions, and habits that positively or negatively affect one’s health status.^1 ^In Lebanon, there is a high prevalence of risky health behaviour. Among the adult population, 38.5% are smokers, 45.8% do not have adequate physical activity, 13.8% have hypertension, 5.9% have diabetes mellitus (DM), 38.0% are overweight, and 27.4% are obese.^2^

Studies have shown better counselling of patients when the doctors themselves have a healthy lifestyle.^3–7 ^The issue of whether or not doctors themselves adopt positive health behaviour has been studied in some countries, with mixed results. However, no such studies were done in Lebanon or the Arab region.

This article presents the results of a preliminary study in Lebanon, which assesses the level of PCPs’ adherence to positive health behaviour, including the implementation of preventive recommendations.

A cross-sectional questionnaire-based survey was conducted among a convenience sample of 227 PCPs attending the Annual Lebanese Family Medicine Conference, held in Beirut in 2014. A 22-item-survey was used, with questions about physicians' demographics and adherence to different components of health behaviour, covering such topics as smoking status, exercise habits, cancer screening, and immunisation compliance.

## Results

### Sociodemographic characteristics

The response rate was 60.4% (137 PCPs), with 51.1% males and 48.9% females. The mean age was 41.3 (±12 years). Twenty-seven (19.7%) reported having a previous medical condition, and 54.0% were affiliated to an academic institution.

### Practices concerning habits and preventive services

#### Lifestyle

Fifty-three physicians (39.0%; missing data [MD] = 1) had ever smoked, while 33 (24.1%) were current smokers. More physicians unaffiliated to academic institutions were current or ex-smokers (*P* = 0.002). In addition, 86 physicians (63.7%; MD = 2) exercised regularly, and 63 (46.0%) were overweight or obese. Significant differences were found between both sexes; more male physicians declared that they were ex-smokers (*P* = 0.018), exercised (*P* = 0.017), and were overweight or obese (*P* = 0.018).

#### Non-communicable diseases

One hundred and thirty (95.6%; MD = 1) physicians had measured their blood pressure (BP) within the past 2 years. Sixty-eight (88.3%) of the eligible physicians had undergone lipid profile screening within the past 5 years, and 93.0% of those eligible for DM screening had undertaken it within the past 3 years.

#### Vaccinations

Eighty-eight physicians (65.2%; MD = 2) had received a tetanus vaccine within the past 10 years, and 69 physicians (50.4%) had been immunised against influenza within the past year. However, only five (12.8%) of the 39 eligible physicians had received the pneumococcal vaccine. Significantly more physicians affiliated to academic institutions were immunised against tetanus (*P* = 0.002) and influenza (*P* = 0.049). Age was found to be the only significant predictor for vaccination compliance after adjusting for academic affiliation, type of practice, and sex using a logistic regression model. For each additional year of age, the odds of taking the influenza vaccine was decreased multiplicatively by 0.94 (95% confidence interval [CI] = 0.902 to 0.980; *P* = 0.03), whereas the odds of taking the tetanus vaccine was decreased multiplicatively by 0.93 (95% CI = 0.894 to 0.977; *P* = 0.03).

#### Cancer screening

As for cancer screening, 14 (37.8%) of the physicians eligible for colon cancer screening had undergone either colonoscopy or fecal occult blood test. Significantly more physicians working in academic institutions had undergone screening for colon cancer (*P* = 0.007). Mammography was undergone by 20 (69.0%) of the eligible female physicians, and Pap smear was undergone by 29 (43.9%) of all female physicians.

### Differences in practices by degree of specialisation

The only statistically significant differences between family physicians and GPs were that a higher number of GPs had smoked in the past (*P* = 0.016), and a greater number of family physicians had been immunised against influenza (*P* = 0.032).

### Substantial barriers

Perceived barriers to adopting positive health behaviour among PCPs were lack of time (72.7%), risks associated with some preventive services and vaccines’ side effects (12.1%), uncertainty of vaccines' effectiveness (10.6%), costs (9.1%), and lack of knowledge (9.9%). Only lack of time was found to be significant when comparing the perceptions of physicians who were working in academic institutions with those who weren't (*P*< 0.001).

## Discussion

This survey showed that PCPs in Lebanon follow the preventive practice guidelines of cardiovascular risk factors, but that there is variable adherence to the other components of positive health behaviour, such as immunisations and cancer screening.

The mean age of PCPs in this study was 41.3 years and 51.1% were males. These results are comparable to those reported in a previous study conducted among Lebanese family physicians, showing an average age of 40.7 years and 56% male participants.^8^

Academic physicians reported better health behaviour, which may be attributed to university hospitals’ policies (for example, a no smoking policy), easy access to screening services, and being 'role models' for residents and staff. Compared to the general Lebanese population, Lebanese PCPs smoked less (24.1% versus 38.5%), exercised more adequately (63.7% versus 54.2%), and were less overweight or obese (46.0% versus 65.4%).^2^ With physicians having a healthier lifestyle than the wider community, they can be role models for their patients with regards adopting positive health behaviour.

There is limited data about this topic internationally and regionally, therefore comparing the Lebanese PCP’s health behaviour with that of other countries is limited. Regarding lifestyle behaviors, compared to the literature, 24.1% of Lebanese PCPs were current smokers, 63.7% exercised adequately, and 46.0% were overweight or obese, versus 1.8%, 58.1%, and 34.7% respectively in US physicians.^3^ On the other hand, PCPs in Lebanon were highly adherent to screening for cardiovascular risk factors, which was in keeping with Western studies with regards to BP and lipid profile measurements.^9,10^ As for vaccination, Lebanese PCPs’ adherence to influenza and tetanus vaccines was similar to the rates reported in the literature.^6,9,11^ However, adherence rate to pneumococcal vaccine was far less than that reported among Israeli PCPs in 2013 (44.5%).^6^

When it comes to cancer screening, the compliance for different types varied. The adherence to colon cancer screening was low in this survey (37.8%); this is slightly better than that reported among PCPs in Spain (24%),^11^ but much worse than that reported among Israeli PCPs (60.9%)^6^ and US physicians (75%).^3 ^As for breast cancer, more than two-thirds of the female physicians in Lebanon screen for it through mammograms, a result that correlates with the literature.^3,6,11^ In the case of cervical cancer, the observed difference in screening between physicians in Lebanon (43.9%) and in Spain (73%)^11^ may be due to some cultural concerns hindering unmarried Lebanese females from having a Pap smear.

There were several limitations in this pilot study. These include selection bias, since the sample was composed only of physicians attending the conference, who may have been more located in the city area, and who may be more academically affiliated; and the use of self-reported data, which cannot be externally confirmed.

Lower age and being affiliated to an academic institution are important predictors of the adherence level of PCPs to positive health behaviour. Improvement in physicians’ adoption of positive health behaviour and adherence to preventive services, particularly in immunisations and cancer screening, is recommended. This study sets grounds for future research to assess PCPs’ beliefs about, knowledge of, and adherence to the preventive recommendations guidelines in Lebanon and the international community. It opens doors for setting interventions to increase the physician’s adherence to different components of positive health behaviour through addressing the main barriers mentioned in this study.
